# From rheumatic diseases to cancer - role of autoantibodies as diagnostic biomarkers

**DOI:** 10.1186/ar3557

**Published:** 2012-02-09

**Authors:** Eng M Tan

**Affiliations:** 1 Department of Molecular and Experimental Medicine, The Scripps Research Institute, La Jolla, CA 92037, USA

## 

Rheumatology has pioneered in the study of autoantibodies by showing that they are not only involved in pathogenesis but are also highly useful as diagnostic biomarkers. The diagnostic biomarker aspect of autoimmunity has gained increasing importance in cancer and many of the insights gained in Rheumatology have contributed to understanding the significance of autoantibodies in cancer.

## Features of autoantibodies in rheumatic disorders

In rheumatic diseases no individual autoantibody-antigen system has sufficient combination of sensitivity and specificity to serve as a useful diagnostic biomarker. Instead, several antigen-antibody systems constructed as profiles of biomarkers are highly effective in distinguishing one disorder from another. In lupus, anti-double strand DNA and anti-Sm distinguishes it from scleroderma, where the profile is anti-DNA topoisomerase 1 and anti-centromere proteins. The autoantigens are cell components involved in universal and basic gene expression pathways, such as Sm in precursor mRNA splicing and DNA topoisomerase 1 in DNA replication and transcription [1].

## Features of autoantibodies in cancer

Autoantibodies in cancer target intracellular molecules referred to as TAAs (tumor-associated antigens). As in rheumatic disorders, no individual autoantibody-antigen system has sensitivity and specificity to serve as a stand-alone diagnostic marker [2]. Most tumors show multiple antibody specificities and with panels of TAA-anti-TAAs (analogous toprofiles) the cumulative sensitivity and specificity reaches diagnostic significance. Different tumorigenesis pathways are activated in similar cell-type tumors from the same organ and are the driving mechanisms behind the autoantibody response. The immune responses are directed to products of oncogenes and tumor-suppressor genes such as p53 and other proteins that regulate and modulate the functions of p53 [3-5].

Protein phosphatase 2A (PP2A) is an important tumor suppressor protein. It is a serine/threonine phosphatase and is a trimeric complex. The B subunit is recruited from several intracellular proteins and the type of B subunit determines the substrate of its tumor suppressor activity. One of the B subunits, p90, was identified in our laboratory with autoantibody from a patient with hepatocellular carcinoma [6]. It was found to co-immunoprecipitate with other subunits of PP2A [7] and was shown to function as an inhibitor of the tumor-suppressor activity of PP2A.

The immune system is capable of sensing dysregulation of tumorigenesis pathways. The goal of continuing research is in developing TAA-anti-TAAs for detecting cancer in individual patients and profiles which are common to specific types of tumors.
